# Nanoscale Hollow Spheres: Microemulsion-Based Synthesis, Structural Characterization and Container-Type Functionality

**DOI:** 10.3390/ma3084355

**Published:** 2010-08-12

**Authors:** Henriette Gröger, Christian Kind, Peter Leidinger, Marcus Roming, Claus Feldmann

**Affiliations:** Institut für Anorganische Chemie, Karlsruhe Institute of Technology (KIT), Engesserstraße 15, 76131 Karlsruhe, Germany

**Keywords:** hollow sphere, nanomaterial, microemulsion synthesis, structural characterization, properties, container functionality

## Abstract

A wide variety of nanoscale hollow spheres can be obtained via a microemulsion approach. This includes oxides (e.g., ZnO, TiO_2_, SnO_2_, AlO(OH), La(OH)_3_), sulfides (e.g., Cu_2_S, CuS) as well as elemental metals (e.g., Ag, Au). All hollow spheres are realized with outer diameters of 10−60 nm, an inner cavity size of 2−30 nm and a wall thickness of 2−15 nm. The microemulsion approach allows modification of the composition of the hollow spheres, fine-tuning their diameter and encapsulation of various ingredients inside the resulting “nanocontainers”. This review summarizes the experimental conditions of synthesis and compares them to other methods of preparing hollow spheres. Moreover, the structural characterization and selected properties of the as-prepared hollow spheres are discussed. The latter is especially focused on container-functionalities with the encapsulation of inorganic salts (e.g., KSCN, K_2_S_2_O_8_, KF), biomolecules/bioactive molecules (e.g., phenylalanine, quercetin, nicotinic acid) and fluorescent dyes (e.g., rhodamine, riboflavin) as representative examples.

## 1. Introduction

Nanomaterials with unusual composition, structure and properties are of general interest to materials science [1,2,3,4]. Amongst nanomaterials exhibiting advanced morphology and structure, nanorods (e.g., metal and metal oxide nanorods) and nanotubes (e.g., carbon nanotubes) are part of comprehensive investigations, as documented by hundreds of papers published each year [5,6,7]. In comparison knowledge on nanoscale hollow spheres is right at the beginning, although as an unusual morphological class of materials hollow spheres have nevertheless evoked manifold interest already. This relates to the large specific surface, the low specific weight, the potentially high mechanical strength and the use of hollow spheres as a container [8]. Initial studies have proven, for instance, that SnO_2_ or Si hollow spheres are promising electrode materials in lithium-ion batteries [9,10], TiO_2_ hollow spheres may serve as very active photocatalysts [11,12], Au, Pt or Pd-filled hollow spheres turn out to be suitable as catalysts and exhibit a certain ability to store hydrogen [13,14,15]. Extraordinary pressure stability was demonstrated with CdS hollow spheres, which—despite a considerable deformation—can withstand shear stresses up to 2.2 GPa [16]. And finally, hollow spheres can be employed as contrast agents in medicine and for encapsulation and transport of inorganic salts, biomolecules or pharmaceuticals [17,18,19]. In many cases, however, the examples mentioned above are currently limited to mesoscaled hollow spheres, *i.e.*, spheres with diameters exceeding 100 nm.

Access to nanoscale hollow spheres is most often performed based on hard-template techniques [8]. As a second experimental approach Kirkendall-type ripening of selected materials allows for hollow sphere formation, too [20,21,22,23]. Finally, a microemulsion-based approach recently came into focus as an alternative strategy to form nanoscale hollow spheres [24]. This microemulsion approach aims to get rid of hard templates in particular, and to allow for high flexibility in synthesis and materials selection. In general, the approach has several beneficial aspects:

(1) Based on equilibrated, thermally stable micelles, the microemulsion approach can be widely modified by adjusting the type and amount of polar/nonpolar phase and surfactants. This allows for fine-tuning the micelle diameter—and as an immediate consequence—controlling the diameter of the resulting hollow spheres.
(2) The possibility to establish water-in-oil-(*w/o*)—as well as oil-in-water-(*o/w*) microemulsions—increases the experimental flexibility of the approach even further, since polar, water-soluble starting materials as well as non-polar ones can be applied according to the specific needs and restrictions of each synthesis.
(3) A wide variety of nanoscale hollow sphere compositions is accessible, ranging from elemental metals to metal oxides, metal sulfides or organic-inorganic hybrids.
(4) While establishing the sphere wall of the hollow shperes, all compounds dissolved inside the micelle are encapsulated. Accordingly, the use of microemulsions gives direct access to container-type functionalities.

With this report we summarize some of our recent results regarding hollow sphere synthesis based on the microemulsion approach. The concept of synthesis is discussed in general and compared to hard-template techniques and Kirkendall ripening. The specific conditions of the microemulsion approach that are required to steer the synthesis of nanoscale hollow spheres are specified. Moreover, current knowledge on the mechanism of hollow sphere formation as well as selected properties of the resulting nanoscale hollow spheres are summarized. The latter aspect especially focuses on container-type functionalities.

## 2. Strategies to Prepare Nanoscale Hollow Spheres

In this section we describe and compare some general aspects of the three most common routes to nanoscale hollow spheres: hard-template based techniques (cf. 2.1), Kirkendall ripening (cf. 2.1) and the microemulsion approach. We will take a closer look at microemulsion techniques that belong to the standard tools to prepare various kinds of massive nanoparticles (cf. 2.2), and that—prerequisite to an essential modification—are quite advantageous with regard to hollow sphere formation, too (cf. 2.3). To round up the potential of microemulsions, their flexibility to obtain different materials as hollow spheres (cf. 2.4), as well as recent approaches to control and fine-tune diameter and cavity size are discussed (cf. 2.5).

### 2.1. Techniques based on hard templates

**Figure 1:**
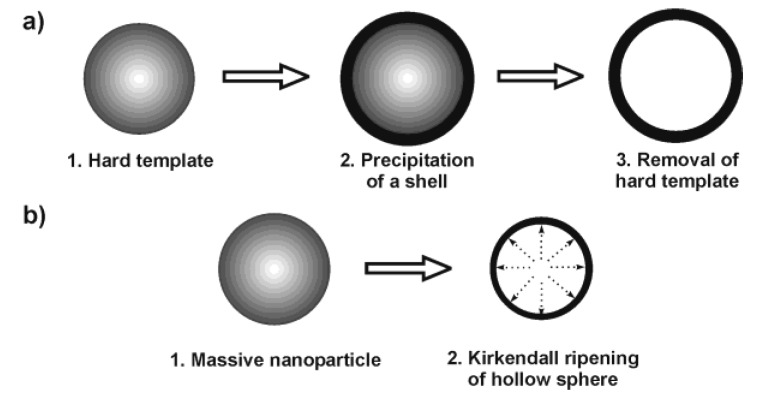
Scheme exploiting the synthesis of hollow spheres via: a) hard-template techniques and b) Kirkendall ripening.

Nanoscale hollow spheres are most often obtained via hard-template methods [8]. Therefore, we discuss some general aspects of this technique first. Accordingly, monodisperse and non-agglomerated nanoparticles—acting as the hard template—are coated with a shell [Figure 1 a)]. Dissolving the template out of the shell as a second step leads to the final hollow sphere. A variety of materials have been used as the hard template, including for instance, Al_2_O_3_, SiO_2_, SnO_2_, TiO_2_, LaF_3_, Co_9_S_8_, Cu_2_S, CuS, CdSe, Ag, Au, Pd or polymer lattices [8,25,26,27,28,29,30,31,32,33]. Removal of these templates is performed, for instance in the case of SiO_2_, by dilute hydrofluoric acid. Metal nanoparticles used as a template can be removed by oxidizing acids. Polymer lattices are burned out at elevated temperature or dissolved with nonpolar solvents. Particularly elegant is the reductive deposition of a more noble metal (e.g., Au) as the shell, while simultaneously dissolving a less noble template (e.g., Pb nanoparticles) [34]. In addition to hard-template techniques, in some cases even massive nanoparticles can be directly converted into nanoscale hollow spheres via so-called Kirkendall ripening [Figure 1 b)] [8,20,21,22,23]. A typical example is related to the conversion of massive hydroxide nanoparticles to oxide hollow spheres.

Although hard-template techniques are widely applied, the availability of monodisperse and non-agglomerated nanoparticles as precursors can be considered as a severe restriction. Moreover, dissolving the template without destroying the newly prepared shell can be complicated. With regard to container-type functionalities hard templates actually turn out to be disadvantageous since the transported agent would have to be introduced inside to the hollow sphere after removing the template.

### 2.2. Microemulsion techniques: some general considerations

**Figure 2:**
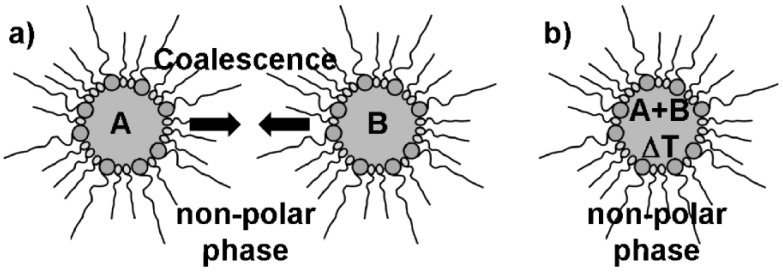
Synthesis of massive nanoparticles via microemulsion techniques with, e.g.,: a) mixing and coalescence of two different *w/o*-micellar systems, each of them containing a starting material A and B; b) thermal initiation of particle nucleation in a single *w/o*-micellar system containing two reactants A and B.

Microemulsion techniques belong to the most common methods to prepare high-quality nanomaterials. This is indicated by hundreds of papers appearing each year and includes numerous nanomaterials, ranging from elemental metals, metal oxides, metal sulfides to organic-inorganic hybrid materials [35,36,37]. Since the underlying equilibrated and thermodynamically stable water-in-oil-(*w/o*)- or oil-in-water-(*o/w*)-micelles exhibit dimensions on the nanoscale (typically 2 to 50 nm in diameter), any particle nucleation inside these surfactant-stabilized droplets—which can be adopted as a nanoreactor—can easily be restricted to the nanoscale, too (Figure 2). Moreover, the resulting nanoparticles are almost perfectly stabilized with concern to uncontrolled growth and agglomeration. This is due to the surfactants that establish the micellar system and as well stabilize the nanoparticles. As a consequence, the overall quality of the resulting nanoparticles in terms of uniformity is normally very high. Much of the adaptability of microemulsion techniques arises from the large number of (commercially) available surfactants. This includes cationic, anionic, non-ionic as well as amphiphilic surfactants. In concrete, cetyltrimethylammonium bromide (CTAB), sodium dodecyl sulfate (SDS), sodium 1,4-bis(2-ethylhexyl)sulfosuccinate (AOT) or polyethylenglycol ethers represent some of the most widely applied surfactants [38]. Quite often co-surfactants are added, typically to reduce the repulsion of similarly charged ionic surfactants. Based on the type and concentration of these surfactants, a precise adjustment and fine-tuning of the micelle diameter is possible. Taking a *w/o*-microemulsion as an example, the micelle diameter basically depends to the surfactant-to-water ratio as given by *ω* = [H_2_O]/[S] (S: surfactant). Since nanoparticles that were prepared in a micelle as a “nanoreactor” often exhibit diameters that directly correlate to the size of the initial micelle, microemulsions in principle allow for size control of nanoparticles “in advance” to preparing them.

Microemulsion synthesis is typically performed by mixing and coalescence of two separate micellar systems—each of them containing at least one reactant [Figure 2 a)] [35,36,37]. When mixing both microemulsions, coalescence of the micelles occurs and initiates the reaction of the starting materials inside the nanosized micellar droplets. Less often all starting materials are incorporated into just one micellar system. In such a case the reaction is initiated by some external trigger, for instance, by increasing the temperature [Figure 2 b)]. A characteristic trait of typical microemulsion techniques is that all the reactants are located in the same phase, *i.e.*, the polar or non-polar phase inside of one or (most often) two different micellar systems. Reactant addition to the dispersant phase as well as to the micelles is rare—but will be an important aspect regarding hollow sphere formation (cf. 2.3).

In sum, the importance and wide application of microemulsion techniques for preparing nanomaterials is very obvious. However, the far majority of these well-established syntheses aims to produce massive nanoparticles [35,36,37]. Microemulsions have been actually already evaluated with regard to their potential to prepare hollow spheres, too. However, the resulting hollow spheres were surprisingly large in diameter (d ≥ 100 nm). This holds especially true when considering the diameter of the initial micelles (d < 30 nm) [39,40]. In other cases the microemulsion approach only resulted in agglomerated nanoparticles or was used to prepare porous solid matrices [41]. Finally, emulsion and miniemulsion systems have been used quite often [42,43,44]. However, the latter systems are thermodynamically non-stable and contain micronsized to even larger droplets. As a consequence, hollow spheres with large diameters (*i.e.*, > 500 nm), broad size distribution and significant agglomeration were obtained.

### 2.3. Nanoscale hollow spheres via the microemulsion approach

**Figure 3:**
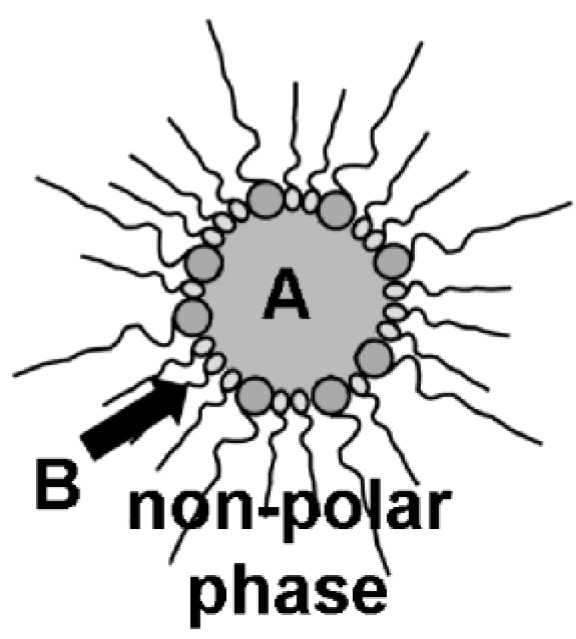
Synthesis of nanoscale hollow spheres via the microemulsion approach at the liquid-to-liquid phase boundary of a *w/o*-microemulsion due to a first reactant (A) inside the polar micellar phase and a second reactant (B) added to the non-polar dispersant phase.

Nanoscale hollow spheres, in principle, can be obtained from microemulsions by a very similar experimental approach as described above for massive nanoparticles (cf. 2.2). In fact, there is only one essential difference: the addition of the starting materials. Thus, a first starting material was located inside the micelle while the second was added to the dispersant phase (Figure 3). Taking a *w/o*-microemulsion as an example, the reaction now proceeds at the liquid-to-liquid phase boundary of the micellar system. This strategy will lead to hollow spheres exhibiting the dimensions of the underlying micelle (*i.e.*, with outer diameters far below 100 nm) if indeed restricted to the phase boundary.

For the synthesis of nanoscale hollow spheres—as presented in the following—most often reversed *w/o*-microemulsions that consist of cetyltrimethylammonium bromide (CTAB) as the surfactant and *n-*hexanol as the co-surfactant are used. Water or 1:1 mixtures of water and methanol are used as the polar dispersant phase; *n-*hexane, *n*-dodecane or toluene constitute the non-polar oil-phase. As a general procedure, a first reactant is dissolved in the water-containing micelle. The formation of an equilibrated and stable *w/o*-microemulsion is indicated by its optical transmittance. Then, the second reactant is slowly added to the non-polar dispersant phase (Figure 3).

To initiate a diffusion-controlled reaction and to strictly limit the formation of a solid to the phase boundary, appropriate experimental conditions are a prerequisite. This includes a suitable type and concentration of the starting materials, a sufficient solubility/non-solubility of the relevant starting materials in the water-/oil-phase, as well as a sufficient reaction rate. In concrete, metal alkoxides, metal triphenylphosphins or metal cyclopentadienids are well suited to be added to the oil-phase; simple metal salts such as halogenides can be used inside the polar phase (Table 1). To control diffusion and reaction rate, the reaction at the phase boundary is most often started—subsequent to the addition of the second precursor—near or slightly below room temperature (0−20 °C) and over a long period of time (2−20 h). Subsequent to the initial formation of a first, very thin solid sphere wall, the reaction rate is sped up by slowly increasing the temperature (from room temperature to 60−80 °C).

**Table 1:** Suitable starting materials for hollow sphere synthesis.

Type of hollow sphere	Starting material for non-polar oil-phase	Starting material for polar water-phase
Au	C_12_H_25_SH	KAuCl_4_
Ag	[Ag(PPh_3_)]_4_NO_3_	NaBH_4_
ZnO	Zn(Cp*)_2_	H_2_O
TiO_2_	TiCl_4_	H_2_O
SnO_2_	Sn(*i-*OC_3_H_7_)_4_	H_2_O
AlO(OH)	Al(*t-*OC_4_H_9_)_3_	H_2_O
La(OH)_3_	La(Cp)_3_	0.1 M KF
Cu_2_S	[Cu(PPh_3_)_2_]Cl	(NH_2_)_2_CS
CuS	Cu(C_9_H_17_COO)_2_	(NH_2_)_2_CS

To separate the nanoparticles from the micellar system, most often destabilization upon addition of a polar, aprotic solvent (e.g., acetone) is applied [35,36,45,46,47,48,49,50]. With this measure, a certain agglomeration of the destabilized particles always occurs, and this is denoted to be a certain drawback of microemulsion techniques. In contrast, the reaction can be also terminated by addition of diethylene glycol (DEG) to the micellar system [51]. This resultes in an immediate phase separation with a top phase that mainly consists of the non-polar dispersant phase and the surfactants, and a DEG bottom phase containing the majority of the hollow spheres. Moreover, DEG is well-known for its efficient stabilization of nanoparticles − an effect that is used here, too [52,53]. Subsequent to centrifugation from DEG, the solid product often can be easily redispersed in DEG, ethanol or water.

**Figure 4:**
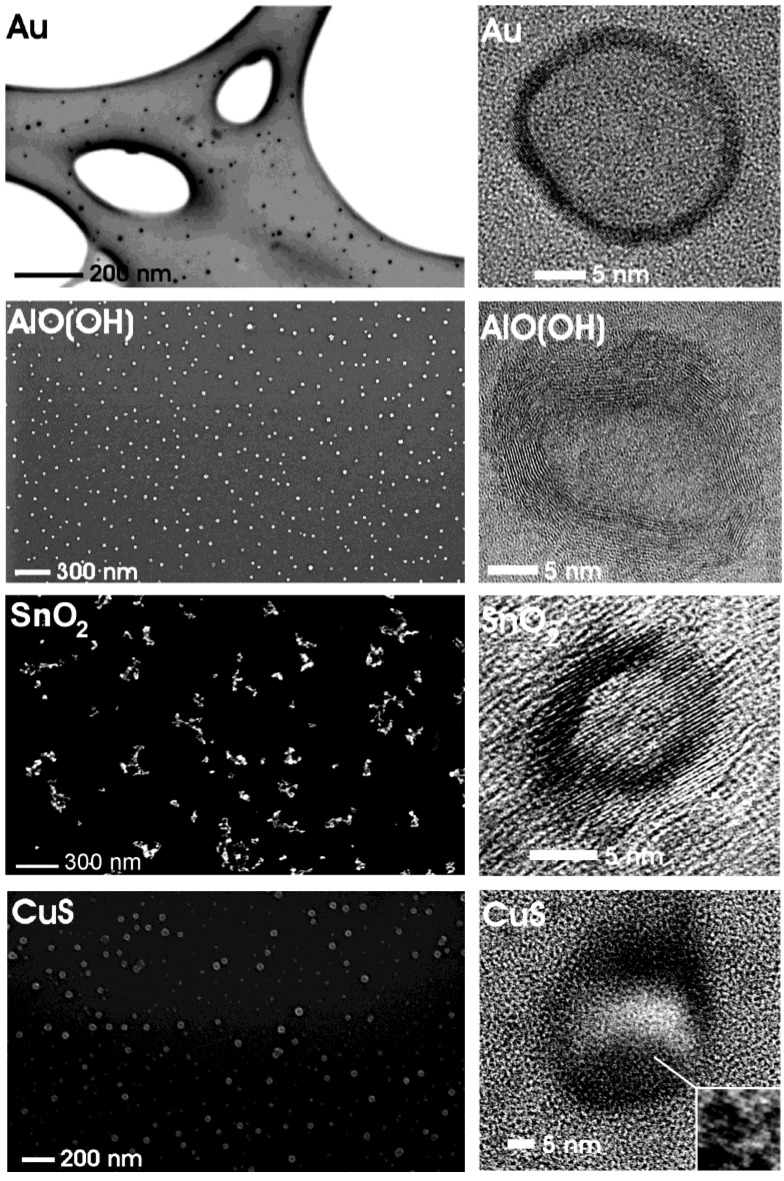
Overview of nanoscale hollow spheres with various compositions gained via the microemulsion approach (modified reproduction from [24]).

Figure 4 gives a first impression of compounds, structures and sizes of nanoscale hollow spheres as obtained via the microemulsion approach. Electron microscopy elucidates morphology and size of these materials (cf. 3.1). According to scanning electron microscopy (SEM) non-agglomerated particles, exhibiting mean diameters of 25 ± 3 nm (Au), 25 ± 6 nm (CuS), 30 ± 5 nm [γ-AlO(OH)] and 20 ± 3 nm (SnO_2_) are obtained. These values are deduced based on a statistical evaluation of at least 100 particles. The presence of hollow spheres that indeed contain an inner cavity is evidenced by high-resolution transmission electron microscopy (HRTEM). To this end, spherical to slightly ellipsoidal particles with outer diameters of about 21−23 nm (Au), 23−32 nm (CuS), 21−25 nm [γ-AlO(OH)], 15−20 nm (SnO_2_), and wall thicknesses of 2−3 nm (Au), 5−11 nm (CuS), 5−7 nm [γ-AlO(OH)] and 3−5 nm (SnO_2_) are prepared.

### 2.4. w/o- and o/w-Microemulsions for hollow sphere generation

**Figure 5:**
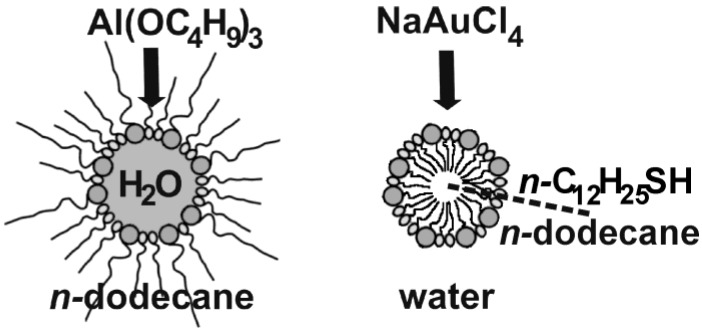
Scheme of hollow sphere formation applying *w/o*- and *o/w*-microemulsions with AlO(OH) and Au as concrete examples.

Additional flexibility of the microemulsion approach with concern to choosing suited starting materials and with regard to the type and composition of the resulting hollow spheres arises from the fact that *w/o-* as well as *o/w*-microemulsions can be used for the synthesis as well [54,55]. This is exploited here for two different types of hollow spheres: AlO(OH) and Au (Figure 5).

In the case of AlO(OH), the synthesis was performed in a *w/o*-microemulsion [55]. In concrete, the micellar system was established by CTAB as the surfactant, 1*-*hexanol as the co-surfactant, *n*-dodecane as the non-polar dispersant phase and a 1:1 mixture of water and methanol as the polar phase inside the micelles. Hollow sphere formation was initiated upon addition of Al(*sec*-OC_4_H_9_)_3_ to the equilibrated, transparent *w/o*-microemulsion (Figure 5). Subsequent to admixing the alkoxide, the micellar system was left to react at room temperature for 12 h. Hence, a simple hydrolysis of the alkoxide occured at the liquid-to-liquid phase boundary. Such a reaction, in principle, could be performed with a lot of metal alkoxides. In detail the following aspects have to be considered to select suitable alkoxides: first, a slow diffusion-controlled hydrolysis is preferred, and second the solubility of the alkoxide has to be much higher in the non-polar than in the polar phase. Subsequent to the reaction, the hollow spheres were separated as discribed in Section 2.3. Detailed characterization of as-prepared AlO(OH) hollow spheres as well as its container-type functionalities will be discussed in the following sections (cf. 3.1, 3.2 and 4.1−4.3).

Au hollow spheres—our second example—were prepared in a *o/w*-microemulsion [54]. Here, the micellar system was established by sodium dodecylsulfate (SDS) as the surfactant, *n-*dodecylthiol as the co-surfactant, demineralized water as the polar dispersant and *n*-dodecane as the non-polar phase inside the micelles (Figure 5). The micellar system was cooled to 10 °C and left to equilibrate for one day. Thereafter, the reaction was started upon addition of NaAuCl_4_℘4H_2_O to the dispersant phase. The yellow colored [AuCl_4_]^−^-containing solution was added slowly and dropwise on a time scale of 10 hours and without stirring. Thereafter the system was again left to react for two days at 10 °C. Herein, *n-*dodecylthiol has two functions: on the one hand, it represents the co-surfactant, and on the other hand it acts as a reducing agent. The latter induces the formation of elemental gold at the phase boundary of the micellar system. For detailed characterization of the resulting Au hollow spheres see Section 3.1 and Section 3.2.

### 2.5. Control of outer diameter and cavity size

**Figure 6:**
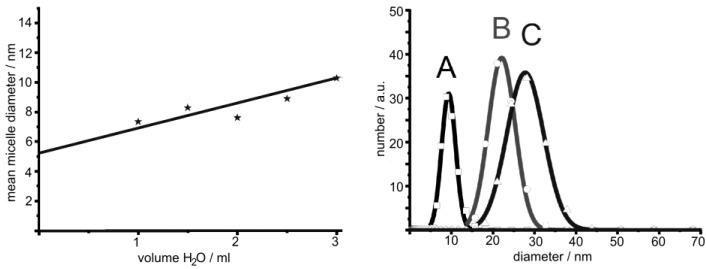
Mean diameter of micelles containing 0.1 M KF-solution in *w/o*-microemulsions consisting of CTAB, *1*-hexanol and *n*-dodecane (left) and size distribution of La(OH)_3_ hollow spheres prepared via microemulsion-based synthesis with different volumes and concentration of aqueous KF solution as the polar phase: A) 1 ml of 0.2 M KF; B) 1 ml of 0.1 M KF; C) 2 ml of 0.1 M KF (modified reproduction from [56]).

As discussed in Section 2.2, in general microemulsions allow for excellent size control of massive nanoparticles. Such size control is most straightforward by adjusting the water-to-surfactant ratio *ω* = [H_2_O]/[S] (S: surfactant) and thereby controlling the micelle diameter. This well-known size control of micelles, in principle, should also allow for controlling the outer diameter and cavity size of nanoscale hollow spheres. This strategy was first evaluated using La(OH)_3_ hollow spheres [56]. Thus, three different micellar systems have been selected with the following composition of the polar phase: A) 1 ml of 0.2 M KF; B) 1 ml of a 0.1 M KF; C) 2 ml of 0.1 M KF. The diameter of the underlying micelles was deduced by dynamic light scattering (DLS) (Figure 6). Subsequent to the addition of lanthanocene as the starting material to the micellar system as well as to hollow sphere formation, centrifugation and purification, the resulting solid was redispersed in diethylene glycol. DLS analysis of the redispersed particles indeed shows an increased mean hydrodynamic diameter with increasing diameter of the initial micelle. Namely this is: 9 ± 4 nm (A), 22 ± 6 nm (B) and 28 ± 9 nm (C) (Figure 6). Obviously, fine-tuning—especially of the very small hollow spheres (< 50 nm)—is indeed possible within the range of stable micelles.

**Figure 7:**
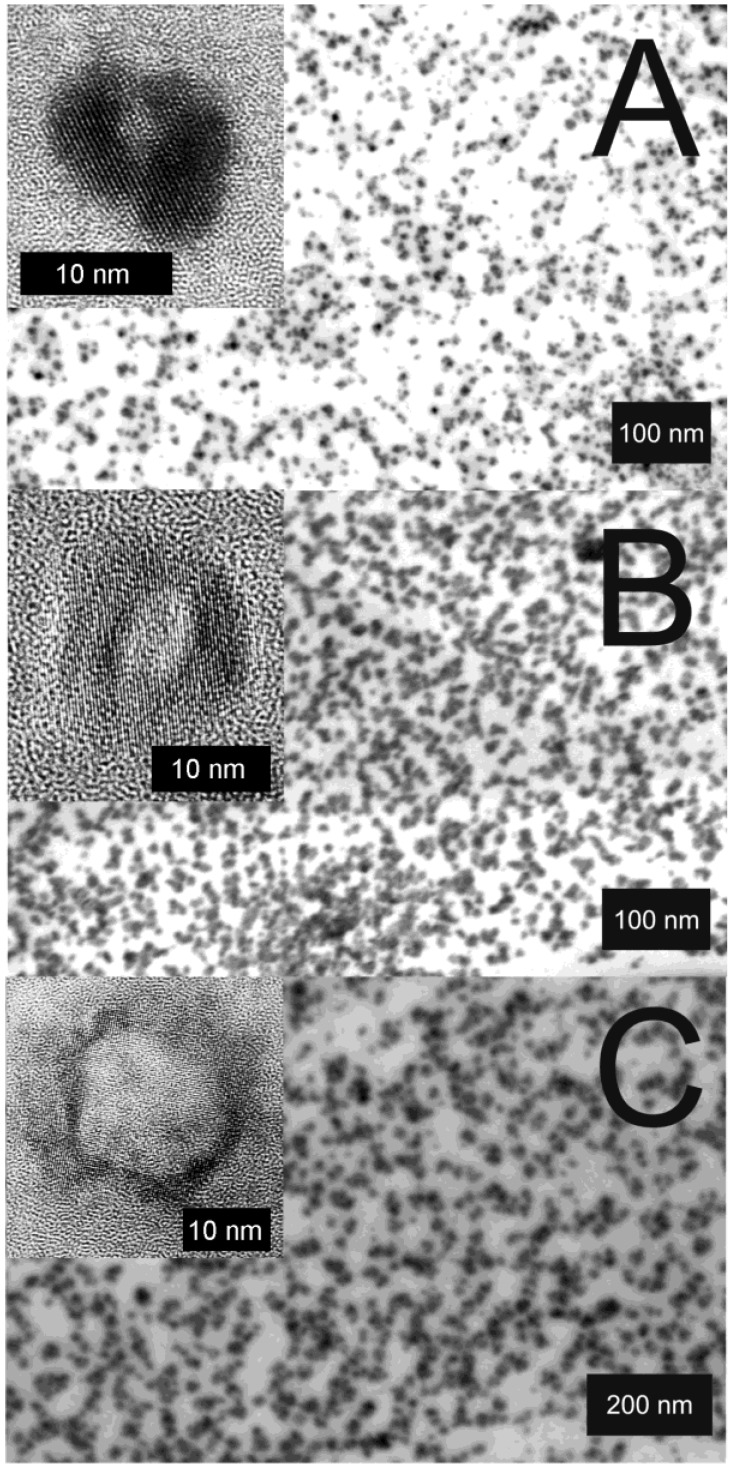
STEM and HRTEM images of La(OH)_3_ hollow spheres prepared in *w/o*-microemulsions with different volumes of the water-phase and different amounts of dissolved KF: A) 1 mL of 0.2 M KF; B) 1 mL of 0.1 M KF; C) 2 mL of 0.1 M KF (reproduction from [56]).

Electron microscopy confirms the diameters stemming from DLS and evidences the presence of hollow spheres (Figure 7). According to STEM and HRTEM images, the hollow spheres exhibit outer diameters of A) 11 ± 3 nm, B) 20 ± 5 nm, C) 30 ± 5 nm and cavity sizes of A) 2 ± 1 nm, B) 7 ± 5 nm, C) 17 ± 5 nm. The thickness of the sphere wall with 4−6 nm in all the cases remains almost similar. While considering the diameter of the non-reacted micelles, its correlation to the cavity size of the hollow spheres becomes obvious. Taking into account that a proceeding growth of the sphere wall in thickness requires a diffusion of the reactants—namely La(Cp)_3_ or H_2_O/OH^−^—through the sphere wall, one can rationalize that this diffusion becomes very slow if the sphere wall reaches a certain size. Consequently, the wall thickness should be *“quasi”* self-terminating, which is in accordance with the experimental observation.

To prepare La(OH)_3_ hollow spheres, aqueous KF was used as the polar phase instead of pure water. This originates from the observation that otherwise only massive La(OH)_3_ nanoparticles were obtained [56]. Comparing hollow and massive nanoparticles, it is quite obvious that the lattice-binding energy favors massive particles instead of the large inner and outer surface of the hollow spheres. In order to nevertheless obtain hollow spheres the ionic strength inside the micelles was increased. This effect is well-known as the *“salting-out effect”* [57]. Thus, addition of KX (X = F, Cl) reduces the solubility of La(OH)_3_ and therefore allows for hollow sphere formation.

## 3. Morphological Characterization

Electron microscopy in all its variations obviously represents the most important tool to prove for the formation of nanoscale hollow spheres. However, additional methods can significantly contribute to summarize the characterization and understanding regarding structure and properties. Out of a wide range of available analytical tools we especially address those to investigate size and size distribution (cf. 3.1) as well as crystallinity and chemical composition of hollow spheres (cf. 3.2). Moreover, the thermal stability and fragility under bombardment with high-energy electrons is discussed (cf. 3.3).

### 3.1. Size and size distribution

Electron microscopy is certainly the most powerful tool to analyze size and size distribution of nanoscale hollow spheres, as well as to prove the presence of an inner cavity. In detail, scanning electron microscopy (SEM), transmission electron microscopy (TEM), high-resolution transmission electron microscopy (HRTEM), scanning transmission electron microscopy (STEM) in both the bright-field (BF) as well as the high-angle anular dark-field (HAADF) mode, and finally selective-area electron diffraction (SAED) are typically applied [58]. However, due to the structural instability of the hollow spheres under typical vacuum and high-energy electron bombardment conditions, these investigations are definitely more than routine (cf. 3.3).

Typical SEM and TEM images of different nanoscale hollow spheres have already been shown in Figure 4. Hereof, SEM images elucidate the presence of non-agglomerated particles with a narrow size distribution. Based on a statistical evaluation of at least 100 particles, a mean particle diameter can be deduced. Accordingly, mean outer diameters ranging from 10 to 60 nm can be obtained for all as-prepared hollow spheres (Table 2). The microemulsion approach in fact allows controlling and fine-tuning of the mean outer sphere diameter by adjusting the size of the initial micelles (cf. 2.5). When aiming at hollow spheres with outer diameters below 10 nm, a formation of massive nanoparticles is typically observed. Outer diameters exceeding 50 nm most often lead to very fragile spheres, so that only destructed fragments of hollow spheres are obtained.

The presence of hollow spheres is evidenced by HRTEM images (cf. Figure 4). Typically the mean inner cavity ranges from 2 to 30 nm (Table 2). Its diameter can be fine-tuned by adjusting the diameter of the initial micelle, too (cf. 2.5). The thickness of the sphere wall remains at a constant value of 2−15 nm (Table 3), which is in accordance with a diffusion-based formation as proposed in Section 2.5. According to HRTEM, the sphere wall normally turned out to be highly crystalline. With a synthesis performed at ambient temperature, this finding is quite surprising, expecially in case of oxides which are normally known to crystallize only at elevated temperatures (> 200 °C) [59]. The observed highly ordered lattice fringes allow one to determine the chemical composition of each single hollow sphere (Table 3).

**Table 2:** Outer diameter and cavity size of various hollow spheres as made via the microemulsion approach. All values given in /nm.

Type of hollow sphere	Mean outer diameter deduced from:		Mean diameter of inner cavity deduced from:	
	DLS	SEM/TEM		STEM/HRTEM
Au	27 ± 4	25 ± 3		~20
Ag	18 ± 3	18 ± 3		~13
ZnO	22 ± 4	20 ± 4		~15
TiO_2_	51 ± 6	48 ± 5		~30
SnO_2_	22 ± 3	20 ± 3		~18
AlO(OH)	33 ± 5	30 ± 5		~22
La(OH)_3_	9 ± 4	11 ± 3		~2
	22 ± 6	20 ± 5		~7
	28 ± 9	30 ± 5		~17
Cu_2_S	41 ± 6	35 ± 5		~20
CuS	25 ± 4	25 ± 6		~27

**Table 3:** Wall thickness and chemical composition as deduced from the observed lattice fringes of various hollow spheres as made via the microemulsion approach.

Type of hollow sphere	Wall thickness deduced from: HRTEM [nm]	Observed lattice fringes along the sphere wall as given by HRTEM [Å]
Au	2−3	2.0 (200); reference with 2.04
Ag	3−5	2.4 (111); reference with 2.36
ZnO	4−6	n.d.
TiO_2_ (anatase)	10−15	3.4 (101); reference with 3.52
SnO_2_	3−5	3.3 (110); reference with 3.35
AlO(OH) (boehmite)	5−7	3.2 (120); reference with 3.17
La(OH)_3_	4−6	2.8 (200); reference with 2.83
		3.2 (101); reference with 3.19
Cu_2_S (chalcocite)	4–8	3.3 (341); reference with 3.37
		3.1 (245); reference with 3.18
CuS (covellite)	5−11	2.0 (110); reference with 1.90

(bulk references obtained from literature data, [60]) (n.d.: no data available yet)

**Figure 8:**
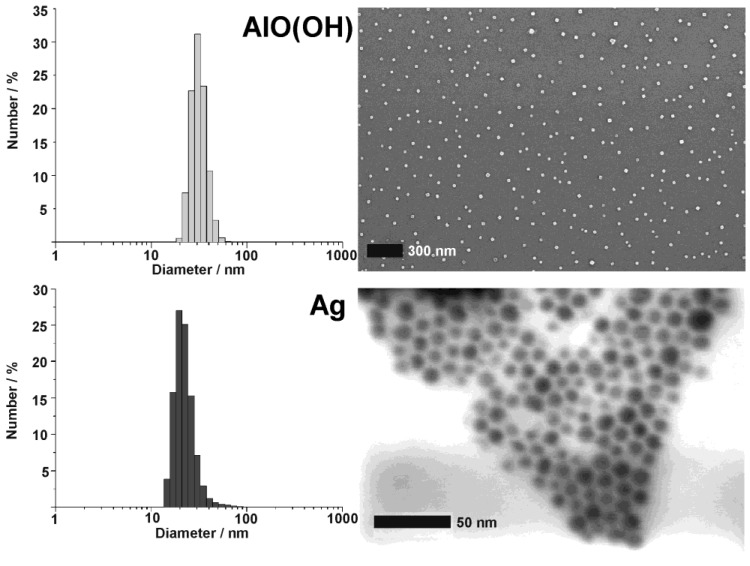
DLS and SEM/STEM overview images showing size and size distribution of as-prepared AlO(OH) and Ag hollow spheres (modified reproduction from [55,61]).

Since all nanoscale hollow spheres were prepared in the liquid-phase, dynamic light scattering (DLS) is an additional beneficial tool to determine size and size distribution as instantaneously obtained after the synthesis (Figure 8). Typically DLS is applied after destabilization of the micellar system, removal of all surfactants and redispersion of the resulting hollow spheres in diethylene glycol or ethanol (cf. 2.3). By comparing the size distribution and mean diameter stemming from SEM and DLS, the primary particle size as well as the degree of agglomeration can be reliably verified. In sum, the interplay of analytical tools that allow for statistical evaluation of a multitude of particles (e.g., DLS, SEM, XRD) and those that allow for a detailed investigation of (some) single particles (e.g., TEM, HRTEM, STEM) result in a powerful and reliable characterization of as-prepared hollow spheres. Such characterization is here shown for AlO(OH) and Ag hollow spheres as representative examples (Figure 8).

### 3.2. Crystallinity and chemical composition

**Figure 9:**
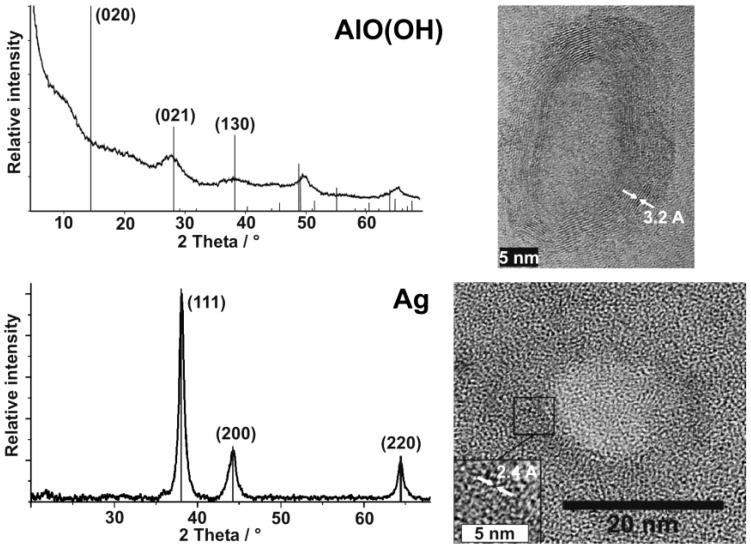
XRD and HRTEM of AlO(OH) and Ag hollow spheres showing crystallinity and composition of a manifold of particles as well as of selected single hollow spheres (modified reproduction from [55,61]).

Particle size, particle size distribution and morphology of the hollow spheres are typically validated by electron microscopy (cf. 3.1). Despite of its importance, electron microscopy nevertheless exhibits an inherent restriction. Thus, only a comparably limited number of particles is analyzed and pictured. Consequently, the obtained results are somewhat limited by statistics in view of the molar quantities of nanoparticles that are present, for instance, in a powder sample. To address this concern, dynamic light scattering is useful to verify size and size distribution of as-prepared hollow spheres in suspension (cf. 3.1). Considering the specific surface, e.g., by the Brunauer-Emmett-Teller (BET) approach, can be an other possibility to verify particle size and prescence of an inner cavity as well [24,53,65]. Aiming at a statistical verification of chemical composition, crystallinity and particle size of as-prepared hollow spheres, furthermore, X-ray powder diffraction pattern, including line-profile analysis can be highly beneficial. This is again illustrated by AlO(OH) and Ag hollow spheres as representative examples (Figure 9).

HRTEM images already pointed to the presence of γ-AlO(OH) and a highly crystalline sphere wall (cf. Table 3). In addition to this analysis of single particles, chemical composition and crystallinity of AlO(OH) hollow spheres are further confirmed by selective area electron diffraction (SAED) [55] as well as by X-ray diffraction pattern. 

Both measures address the manifold of particles of a powder sample (Figure 9). With regard to AlO(OH), XRD powder diffraction pattern are in accordance to reference data [55,60], however, it has to be considered that the sphere wall exhibits only a weak scattering power. All Bragg peaks observed are therefore broad and with low intensity. Moreover, the crystalline sphere wall does definitely not match with the conditions of an isotropic single crystal. Thus, the intensity of reflections can be different from the bulk-phase. With regard to the AlO(OH) hollow spheres, it is most remarkable that the (020) Bragg peak is not observed (Figure 9). This finding can be explained by two arguments. On the one hand, the [020] lattice plane is tilted out of the plane of the sphere wall (which crystallizes with [120], cf. 3.1). Consequently, the scattering power of this lattice plane is significantly weakened. Moreover, for high-pressure modifications of boehmite the intensity of (020) is known to be almost zero. In the case of the hollow spheres, the curvature of the crystalline wall might lead to a certain “internal pressure” as it was observed already for carbon shells as well as for SnO_2_ hollow spheres [62,63,65].

Silver hollow spheres—as our second example—exhibit outer particle diameters of 15−20 nm and a sphere wall, 3−5 nm in thickness according to SEM and TEM images (cf. 3.1, Table 2). HRTEM images show as-prepared hollow spheres to be crystalline and with highly ordered lattice fringes. The observed d-values with 2.4 Å match well with the (111) lattice plane of elemental silver (cf. 3.1, Table 3). X-ray powder diffraction (XRD) pattern confirm the chemical composition and crystallinity—obtained for single particles via electron microscopy—also for a manifold of particles of a powder sample (Figure 9). Moreover, the lattice parameter of a = 409.0 ± 3 pm was determined from XRD peak positions, and is in good agreement with a = 408.5 pm of face-centered cubic bulk-silver [60]. Again, the reflections of the hollow spheres are significantly broadened. This points to small crystallite sizes and is well in agreement with the shell-like structure. Line shapes of all Bragg peaks were here derived via Voigt-functions and used to determine the microcrystalline properties [64]. Thus, an average crystallite size of 9 nm along (111) and (110), and of 6 nm along (001) was calculated for the hollow spheres from the single-line integral width. These data are well in agreement to DLS, STEM and HRTEM (cf. Figure 8 and Figure 9).

Altogether, a reliable characterization of as-prepared nanoscale hollow spheres, including outer diameter, cavity size and wall thickness, chemical composition and crystallinity requires different and independent analytical tools such as DLS, SEM, STEM, TEM, HRTEM and XRD. Single particles have to be analyzed with high resolution on the one hand. On the other hand, statistical information addressing the huge manifold of particles has to be gained. Besides the analytical methods described here, additional information can be obtained from infrared spectroscopy (FT-IR), nuclear magnetic resonance (NMR) spectroscopy, fluorescence spectroscopy (FL), thermogravimetry (TG), small-angle X-ray scattering (SAXS), X-ray absorption spectroscopy (XAS, XANES, EXAFS), Mößbauer spectroscopy and many other methods that have been used and described elsewhere [24,54,55,56,57,58,59,60,61,65,71].

### 3.3. Stability at elevated temperatures and under electron bombardment

All nanoscale hollow spheres turn out to be highly fragile and very sensitive to electron bombardment with effects ranging from a partial collapse of the sphere structure to a complete disappearance of the hollow spheres. To this concern, as-prepared hollow spheres are first of all filled with water stemming from the microemulsion approach (cf. 2.3). The consequence of this water filling can be illustrated, for instance, with SnO_2_ hollow spheres exhibiting an outer diameter of 15−25 nm and an inner cavity of 10−20 nm in diameter [65]. If these as-prepared SnO_2_ hollow spheres were just centrifuged from suspension and left to dry in air at room temperature, thermogravimetry of the resulting dry powders indicates a weight-loss of as much as 18% up to 400 °C due to slow release of water encapsulated inside the inner cavity. If such water-loaded non-dried hollow spheres were to be placed under vacuum (e.g., in an electron microscope), rapid evaporation of water would have caused their complete destruction. Therefore, careful and slow drying is required with precise adjustment of temperature (e.g., by increasing from room temperature to 60 °C within 12 h) and pressure (e.g., by decreasing from ambient pressure to 10^2^ Pa within 24 h). If such a drying procedure was applied with the above SnO_2_ hollow spheres, thermogravimetry thereafter shows a weight-loss of <5% only up to 400 °C, and the hollow sphere morphology remaining intact [24,65].

**Figure 10:**
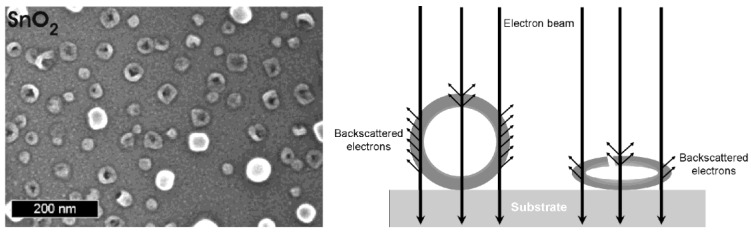
SEM images of partly destroyed SnO_2_ hollow spheres with a scheme exploiting the different contrast and charging of destroyed and intact hollow spheres (reproduction from [24]).

In addition to the above considerations regarding careful drying, even the dried hollow spheres turned out to be highly sensitive to electron beams. Consider that the current density is increased at high magnification (200−300 kV) to typical values of up to 100 A cm^−2^ [66]. The incoming energy due to electron bombardment and charge transport leads to local heating as well as to significant charging of the samples. As a consequence, collapse and complete destruction is often observed on a time scale of some seconds. This situation is best visulalized in case of hollow spheres with comparably large diameters. In opposite to the above mentioned example, SnO_2_ is here used with outer diameters of 40−60 nm (Figure 10) [24]. Intact spheres are indicated by comparably high charging effects as well as by a comparably high contrast. Collapsed spheres behave like empty ballons. The remaining flat structures show significantly lower charging—and due to the decreased number of backscattered electrons—a low contrast. Perforation of the sphere wall is partially visible, too.

**Figure 11:**
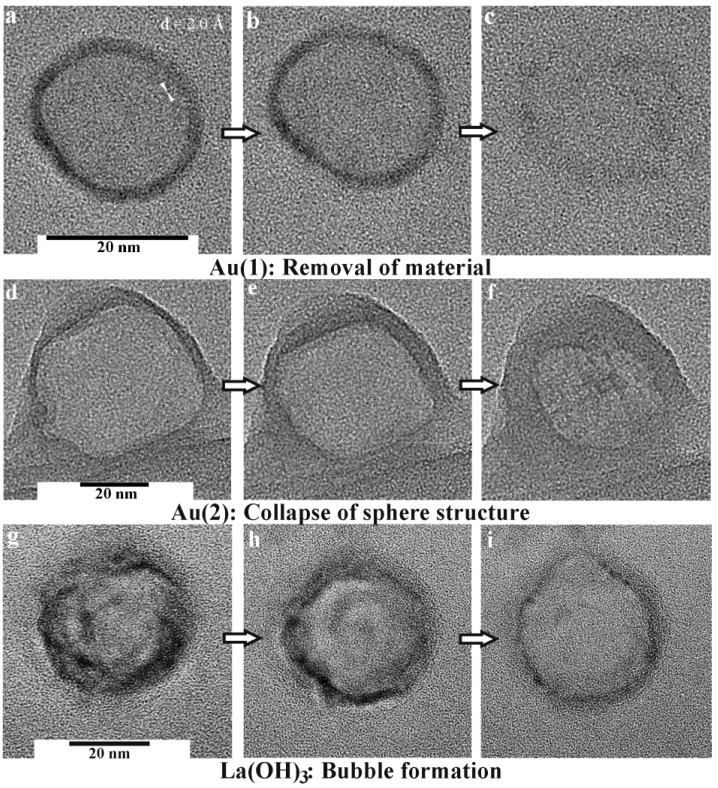
Destruction of hollow spheres under TEM conditions: a−c) Au, 15−20 nm in diameter; (d−f) Au, 40−60 nm in diameter; (g−i) La(OH)_3_, 30−35 nm in diameter (modified reproduction from [54,56]).

Hollow spheres exceeding diameters of 40−50 nm—subject to careful drying and removal of all water stemming from the microemulsion—are reasonably stable under TEM conditions. However, the smaller the diameter of the hollow spheres, the more complicated the TEM analysis at high-resolution is. In some cases destruction of the hollow spheres can follow after electron microscopy. This is most impressively illustrated with differently sized gold hollow spheres, namely, mean outer diameters of 15−20 nm and 40−60 nm. The thickness of the wall in both cases is almost similar (2−5 nm) [54]. Here, two paths of destruction seem to occur: a first destruction process due to complete removal of gold in the case of the smaller hollow spheres (Figure 11a−c), and a partial collapse of the sphere wall in the case of the larger ones (Figure 11d−f). Both these effects occur on a time scale of some seconds.

Especially in the case of gold, it is well-known that even massive gold particles are quite unstable under illumination by a high-energy electron beam. The underlying phenomena such as particle movement, structural change and deformation have been studied in detail for two decades [67]. Massive structural rearrangements are facilitated by a *“gas”* of highly mobile atoms which was observed directly by HRTEM on the surface of Au nanoparticles [68]. The change of cluster shapes can be explained by the concept of *“quasi-melting”* [69]. The *“quasi-molten”* state is observable over time scales which are well accessible in a transmission electron microscope if dissipation of the excess energy is impeded for small contact areas of the particle to the substrate. Williams has suggested that local heating can be understood by the production of Auger electrons [67]. The (partial) dissipation of the energy of Auger electrons in the particles may lead a local temperature increase of up to 2,100 °C whereby the melting point of gold (m.p.: 1,064 °C) is greatly exceeded. The dissipation of the excess energy was suggested to take place on a time scale of 10^−12^ s, which prevents massive evaporation, but it may be the origin of the gas of highly mobile Au atoms on the cluster surfaces. In the case of the hollow spheres, similar processes can be assumed to take place. The effect of dissolution was observed already in case of small solid gold clusters, and has been ascribed to Ostwald ripening of an ensemble of particles with a finite size distribution [70].

In sum, destruction of nanoscale gold hollow spheres under bombardment with high-energy electrons is accompanied by two processes: (1) the presence of a gas of highly mobile Au atoms on the sphere surface facilitates a complete vanishing of small hollow spheres (Figure 11a−c). Deconstruction of these hollow spheres then results in a temporal recondensation of small diffusing Au species and finally yields very small massive nanoparticles, about 1−5 nm in diameter. (2) Deposition of energy by high-energy electrons and its dissipation will affect larger hollow spheres in a different way. Here, mobile Au atoms on the surface reduce the effective solid thickness of the sphere wall, which contributes to the destabilization of the structure. High-temperature excursions by the excitation of Auger electrons generate phonons, which subsequently lead to a collapse of the hollow spheres (Figure 11d−f). This structural collapse results in a formation of thin gold platelets exhibiting the overall diameter of the former hollow sphere. Typically, these residual nanoparticles and platelets are crystalline, but often exhibit different lattice planes [*i.e.*, (111) with d = 2.4 Å] as compared to the initial hollow spheres [*i.e.*, (200) with d = 2.0 Å] [54].

Although the melting point of metal oxides is much higher compared to metals, oxide hollow spheres turned out to be highly sensitive to electron-beam induced damage, too. In the case of La(OH)_3_, for instance, a series of TEM images of selected hollow spheres at first shows a slight growth in diameter (Figure 11g−i) [56]. Next, a kind of a bubble formation is observed, that is followed by complete destruction of the sphere wall. All this happens again on a time scale of some deciseconds to seconds. Considering a local heating of the spheres due to the electron beam, this observation can be attributed to an initial dehydration of the La(OH)_3_ sphere wall, which is accompanied by a certain internal water vapor pressure. The latter causes the bubble-type protuberance, and finally the destruction of the hollow sphere. Thermogravimetry confirms this view since thermal decomposition of La(OH)_3_ with evaporation of water occurs with a massive and continuous weight-loss from 200 °C up to 600 °C. If dehydration at ambient pressure starts at 200 °C, a dehydration occuring under TEM and full-vacuum conditions is not a surprise.

**Figure 12:**
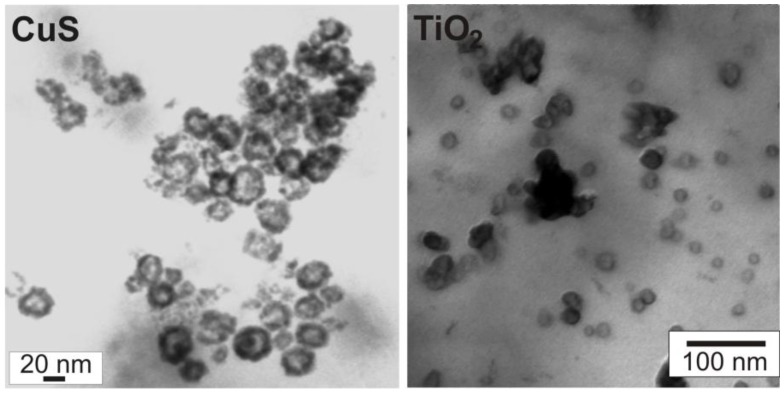
Scanning transmission electron microscopy (STEM) of CuS and TiO_2_ hollow spheres [71].

To overcome the inherent instability of as-prepared nanoscale hollow spheres, scanning transmission electron microscopy (STEM) can be a useful option. Here, the acceleration of the electron beam can be reduced to about 20−30 kV as compared to 200−300 kV for standard TEM analysis. By this technique the presence of the inner cavity can be reliably proven on the one hand, and on the other hand, a statistically relevant number of particles is pictured—ranging between SEM images displaying several tens to hundreds of particles and TEM images with only a few particles visible on one image. As an example, STEM images of CuS and TiO_2_ hollow spheres are shown here (Figure 12). To differentiate between sphere wall and inner cavity at lower acceleration voltages, monoparticulate layers are prerequisite. This needs careful preparation of suited specimens for STEM analysis. Aiming at chemical analysis and crystal structure of the as-prepared hollow spheres, however, HRTEM is indispensable (cf. 3.1, 3.2).

## 4. Container-Type Functionalities

The adaptability of the microemulsion technique and its flexibility regarding the composition of the sphere wall can be considered as a special strength of the experimental approach. However, it turned out to be even more flexible when aiming at container-type functionalities of nanoscale hollow spheres. In principle all kinds of *“transport agents”*—with hydrophilic and hydrophobic features—can be dissolved in *w/o-* or *o/w-*microemulsions prior to establishing the sphere wall. In contrast to the most common concepts [25,26,27,28,29,30,31,32,33], moreover, no template has to be removed. Aiming at drug delivery, on the other hand, large-volume hollow spheres (e.g., polyelectrolyte capsules) with diameters of 500 nm up to several microns are well-known. These materials, however, follow separate concepts of synthesis and exhibit different material properties (e.g., regarding size and volume, elasticity and fragility, osmotic pressures and capillary forces) [72,73,74].

**Figure 13:**
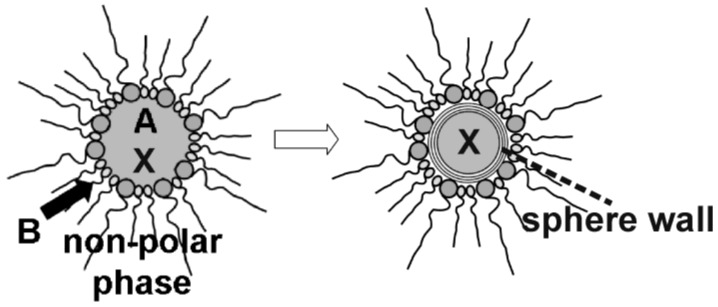
Scheme showing the encapsulation of compounds (X) prior to hollow sphere formation via reaction of the starting materials (A and B) at the liquid-to-liquid phase boundary of *w/o*-micelles.

In sum, the microemulsion approach may offer a very flexible access to uniform nanocontainers as well as to a facile filling process (Figure 13). In the following, encapsulation and release of inorganic salts (cf. 4.1), biomolecules and bio-active molecules (cf. 4.2) as well as of fluorescent dyes (cf. 4.3) is summarized. Note that we indicate all filled hollow spheres as filling@sphere, following the notation of endohedral fullerenes [75].

### 4.1. Encapsulation of inorganic salts

**Figure 14:**
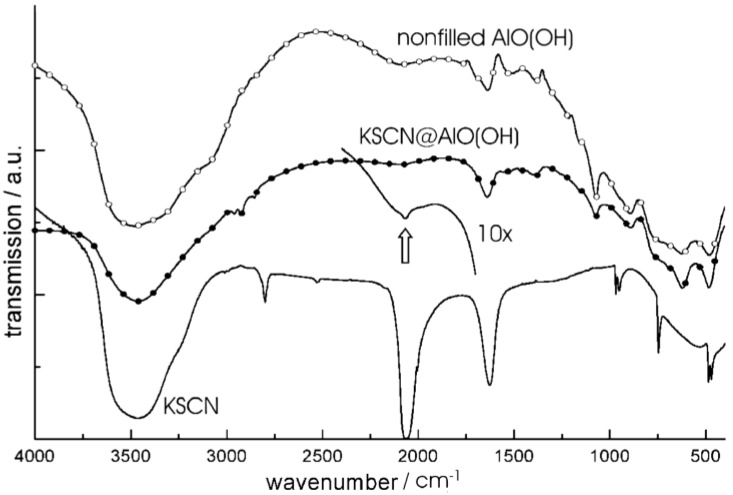
FT-IR spectra of KSCN@AlO(OH) with the vibration of [SCN]^−^ indicated by arrows (spectra obtained with 1 mg and 10 mg (10x) of sample per 400 mg of KBr; spectra of nonfilled AlO(OH) hollow spheres and pure KSCN as references; modified reproduction from [24]).

The container-type functionality of nanoscale hollow spheres is first validated based on the encapsulation of typical inorganic salts, namely KSCN, K_2_S_2_O_8_ and KF. These compounds have been selected since their presence can easily be detected by qualitative and quantitative means. With regard to synthesis, all inorganic compounds have been first dissolved inside the aqueous micelle prior to establishing and closing the sphere wall (cf. 2.4). Their presence—subsequent to hollow sphere synthesis and certain washing processes—is than investigated spectroscopically as well as by chemical analysis. Figure 14 shows FT-IR spectra of nonfilled AlO(OH) hollow spheres, KSCN-filled spheres [KSCN@AlO(OH)] and pure KSCN as a reference [24]. Note that the absolute concentration of KSCN inside the sphere is comparably low [1 mg of KSCN@AlO(OH) and 400 mg of KBr per pellet used for FT-IR]. As a consequence, the relevant vibrational bands are low in intensity, too. By first sight, the spectra of KSCN@AlO(OH) are very comparable to those of the nonfilled spheres [ν(O-H): 3,800−3,000 cm^−1^, δ(H_2_O): 1,650−1,550 cm^−1^]. A closer look, however, clearly indicates the most intense vibration of KSCN [ν_as_(SCN): 2,100−2,050 cm^−1^]. This holds especially true if spectra of KSCN@AlO(OH) were recorded with an increased amount of sample to guarantee for sufficient intensity [10x indicates 10 mg of KSCN@AlO(OH) per 400 mg KBr].

**Figure 15:**
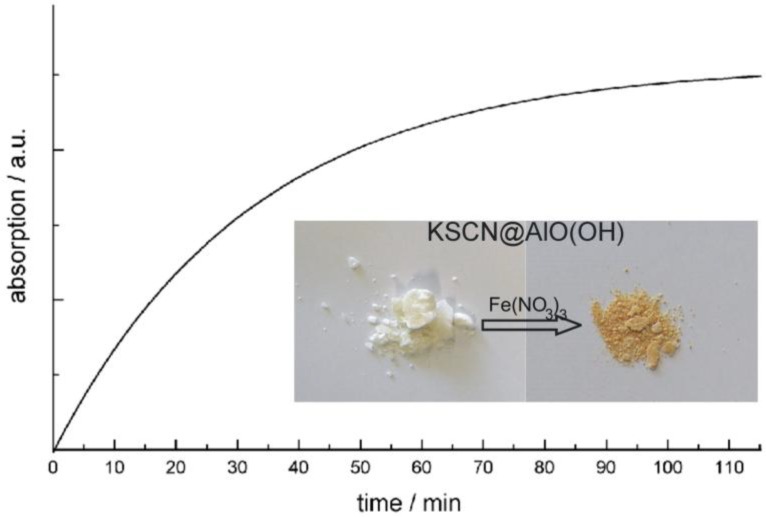
Photos of KSCN@AlO(OH) before and after reaction with Fe(NO_3_)_3_ and time-dependent increase of Fe(SCN)_3_ absorption (at 470 nm) during the reaction (modified reproduction from [24]).

In addition to FT-IR spectra, the presence of KSCN is validated by means of classical qualitative analysis, namely by addition of aqueous Fe(NO_3_)_3_ [76]. Thus, the characteristic reddish color of Fe(SCN)_3_ is clearly visible (Figure 15). The reaction was quantified by optical spectroscopy. UV-VIS spectra of KSCN@AlO(OH) prior and subsequent to the reaction with Fe(NO_3_)_3_ as well as Fe(SCN)_3_ as a reference, confirm the formation of Fe(SCN)_3_. When comparing the product of the reaction and the reference, the spectra differ to some extent. However, it should be considered first that the reference is not a nanomaterial, and second that the product of the reaction consists of a mixture of reddish Fe(SCN)_3_ and colorless AlO(OH). Finally, the course of reaction was followed by recording the time-dependend increase of the Fe(SCN)_3_ absorption (Figure 15). While the reaction in water—upon addition of Fe^3+^ and SCN^−^—only takes some seconds, a duration of more than 60 min is observed here. Considering the required diffusion of reactants through the sphere wall, such a finding is in accordance to the expectation.

**Figure 16:**
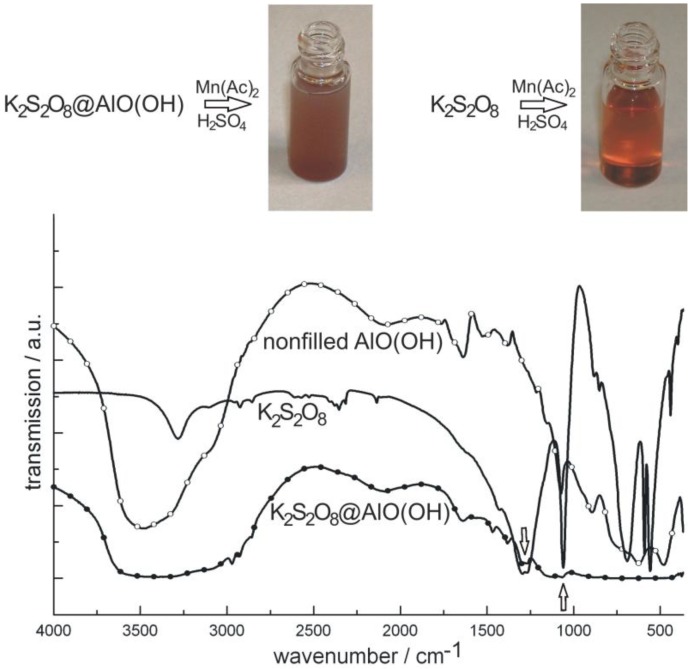
Photos of K_2_S_2_O_8_@AlO(OH) before and after reaction with MnAc_2_ (similar reaction with pure K_2_S_2_O_8_ as a reference) and FT-IR spectra of K_2_S_2_O_8_@AlO(OH) (with nonfilled AlO(OH) hollow spheres and pure K_2_S_2_O_8_ as references).

As a second example of container-type functionalities, the encapsulation of K_2_S_2_O_8_ in AlO(OH) hollow spheres [K_2_S_2_O_8_@AlO(OH)] is shown. In brief, 100 mg K_2_S_2_O_8_ were encapsulated in about 120 mg of AlO(OH) hollow spheres. K_2_S_2_O_8_ as a strong oxidizing agent was again dissolved inside the aqueous micelle prior to establishing and closing the sphere wall (cf. 2.4). After separation and washing of the hollow spheres, the prescence of K_2_S_2_O_8_ was qualitatively proven upon addition of Mn(Ac)_2_ and few drops of concentrated H_2_SO_4_ [76]. By this measure, first of all, the sphere wall was dissolved. Thereafter, Mn^2+^ is oxidized by [S_2_O_8_]^2−^ to form [MnO_4_]^−^. The latter can be easily recognized by its deep reddish-violet color (Figure 16). As a reference similar reaction was performed with pure K_2_S_2_O_8_. Finally, the presence of K_2_S_2_O_8_ is evidenced by FT-IR spectra that clearly show the most intense vibrations of [S_2_O_8_]^2−^ in K_2_S_2_O_8_@AlO(OH) (Figure 16).

**Figure 17:**
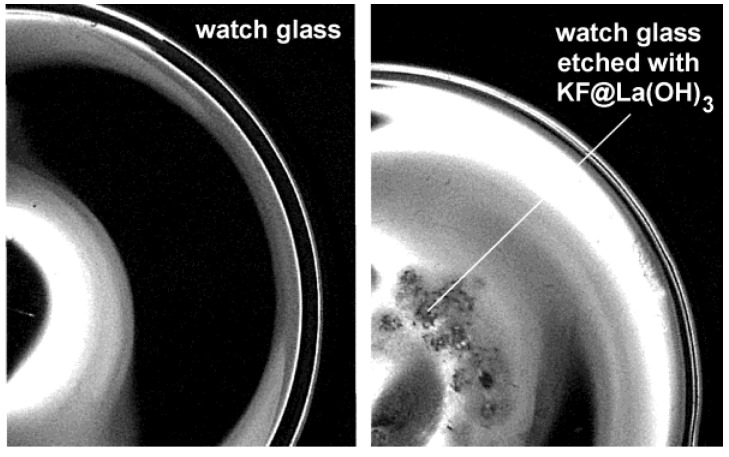
Watch glass prior and subsequent to etching with KF@La(OH)_3_ (reproduction from [56]).

For our last example on the encapsulation of inorganic salts, we pick up the formerly discussed KF-loaded La(OH)_3_ hollow spheres [KF@La(OH)_3_] (cf. 2.5) [56]. KF (or KX with X: halogenide) was here used to increase the polarity of the aqueous micelle. For La(OH)_3_ only this measure allows for preparing hollow spheres instead of massive nanoparticles. The presence of KF was here confirmed, on the one hand, by thermal decomposition of as-prepared KF@La(OH)_3_. Surprisingly, thermogravimetry (TG) proceeds with a massive and continuous weight-loss (~20 wt %) at 200−600 °C with LaOF as the TG-remnant. This finding is in opposite to the decomposition of bulk-La(OH)_3_ (with La_2_O_3_ as the product of thermal decomposition) and proves the presence of molar amounts of the fluoride. Note that molar amounts of aqueous KF (0.1−0.2 M) have been provided inside the micellar system prior to the formation of La(OH)_3_ as the sphere wall (cf. 2.5). Encapsulation and presence of KF in KF@La(OH)_3_ can be further validated by dissolving La(OH)_3_. Thus, upon depositing some KF@La(OH)_3_ on a watch glass and subsequently adding of a few droplets of concentrated hydrochloric acid, etching of the glass surface due to HF formation is observed (Figure 17).

### 4.2. Encapsulation of biomolecules and bioactive molecules

Following the concept of encapsulation of inorganic salts inside nanoscale hollow spheres, biomolecules or bioactive molecules can be similarly encapsulated via the microemulsion approach (cf. 2.5, 4.1). To this end, phenylalanine, quercetine and nicotinic acid were selected due to their biomedical relevance. 

**Figure 18:**
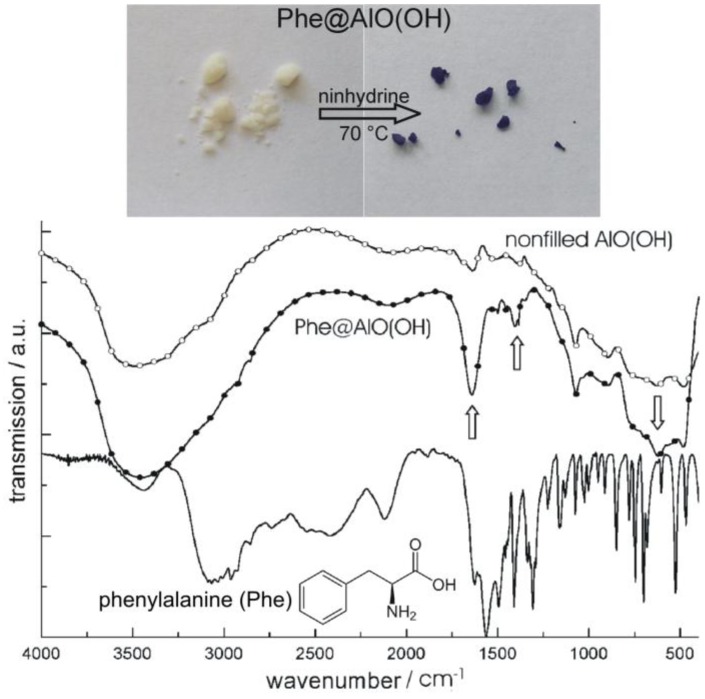
Photos of Phe@AlO(OH) before and after reaction with ninhydrine and FT-IR spectra of Phe@AlO(OH) (vibrations of Phe indicated by arrows; nonfilled AlO(OH) hollow spheres and pure phenylalanine as references; modified reproduction from [24]).

**Figure 19:**
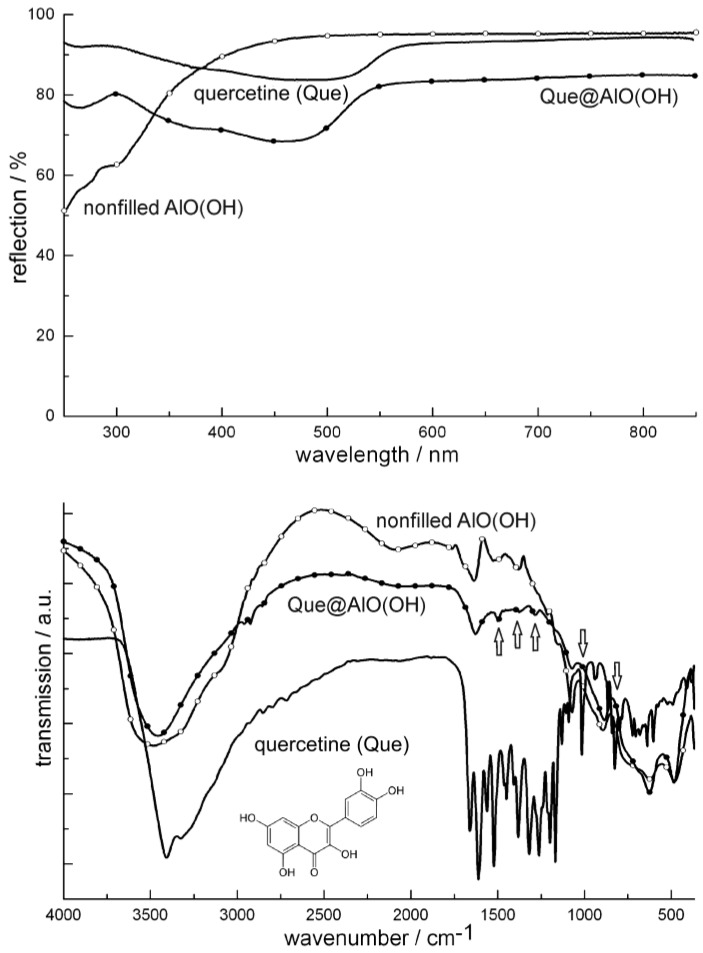
UV-Vis and FT-IR spectra of Que@AlO(OH) (vibrations of quercetin indicated by arrows; nonfilled AlO(OH) hollow spheres and pure quercetine as references).

We first describe the encapsulation of the aminoacid phenylalanine (Phe) [77]. As described before, Phe was dissolved inside the aqueous micelles prior to establishing AlO(OH) hollow spheres [24]. Its presence was again confirmed based on FT-IR spectra while comparing nonfilled AlO(OH) hollow spheres, Phe-filled spheres [Phe@AlO(OH)] and pure Phe as a reference (Figure 18). Like phenylalanine, the flavonol and anti-tumor agent quercetin (Que) as well as nicotinic acid (Nic)—known as niacin or vitamin B3—were encapsulated in AlO(OH) hollow spheres (cf. 2.5) [77]. Thus, 100 mg of quercetin and 50 mg of nicotinic acid were encapsulated in about 120 mg of AlO(OH) hollow spheres. The successful encapsulation is again evidenced spectroscopically. Thus, UV-Vis- and FT-IR spectra of quercetin-filled hollow spheres [Que@AlO(OH)] are compared in Figure 19 to nonfilled AlO(OH) hollow spheres and pure quercetin as references. 

**Figure 20:**
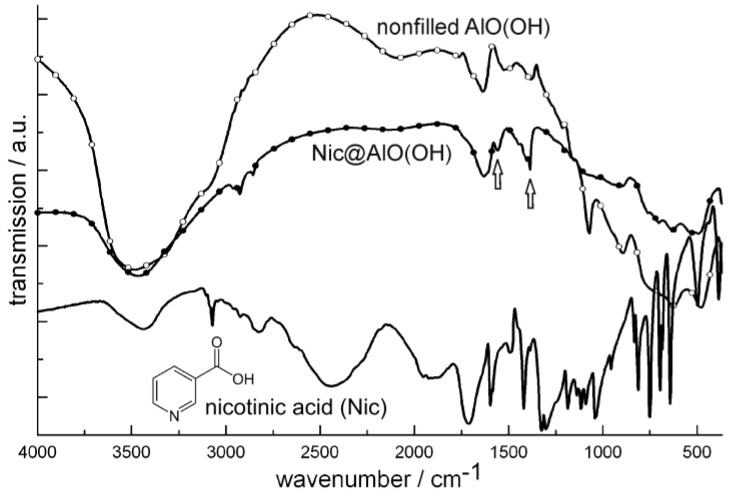
FT-IR spectra of Nic@AlO(OH) (vibrations of nicotinic acid indicated by arrows; nonfilled AlO(OH) hollow spheres and nicotinic acid as references).

Figure 20 similarly shows FT-IR spectra of niacin-filled hollow spheres [Nic@AlO(OH)] as compared to nonfilled AlO(OH) hollow spheres and pure nicotinic acid. FT-IR spectra indicate the most intense vibrations of quercetin and nicotinic acid also to be present after encapsulation in AlO(OH) (Figure 19 and Figure 20). The relevant vibrations are weak in intensity due to the overall low amount of Que and Nic. Since AlO(OH) shows strong absorption by itself, Que and Nic can be detected best by direct comparison to FT-IR spectra of nonfilled hollow spheres. In addition, quercetin can be easily recognized by its bright orange color that is even visible with the naked eye in Que@AlO(OH). UV-Vis spectra quantify this color and show the expected characteristic absorption between 300 and 550 nm (Figure 19).

### 4.3. Encapsulation of fluorescent dyes

**Figure 21:**
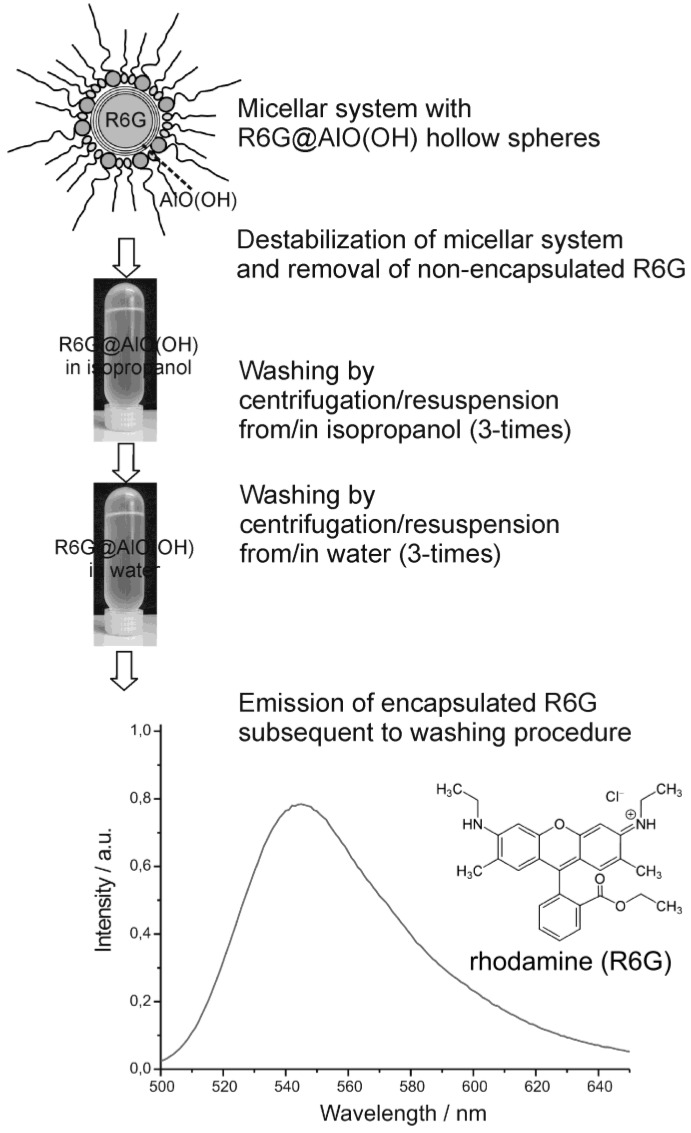
Proving the fluorescence of R6G@AlO(OH) subsequent to the destabilization of the micellar system and advanced washing procedures performed by centrifugation/resuspension from/in isopropanol as well as from/in water (λ_excitation_ = 480 nm).

Finally we discuss the encapsulation of fluorescent dyes in nanoscale hollow spheres. The intense emission of these dyes can be used for their straightforward detection, even in very low concentrations. Accordingly, fluorescent dyes may serve as useful test objects to study mechanistic aspects of encapsulation and release. As a first example, rhodamine (R6G) was encapsulated inside AlO(OH) hollow spheres [R6G@AlO(OH)]. Its intense fluorescence thereafter was recorded by fluorescence spectroscopy [55]. In detail, the hollow spheres were filled with an aqueous 10^−7^ M solution of R6G. During centrifugation and washing R6G@AlO(OH) was controlled by photoluminescence for non-controlled release of R6G (Figure 21). Thus, R6G remaining in the supernatant was only detected subsequent to the very first centrifugation. This finding is ascribed to minor amounts of R6G remaining outside of the hollow sphere during sphere wall formation. To remove all constituents of the micellar system, as-prepared hollow spheres were carefully purified and washed, namely, by resuspending/centrifugation three times in/from isopropyl alcohol, followed by resuspending/ centrifugation three times in/from water. These sequential steps of resuspension and centrifugation of R6G@AlO(OH) on a time scale up to 60 min did not show any additional release of R6G to the supernatant. Finally, R6G@AlO(OH) was resuspended in water. This final suspension shows intense photoluminescence, which evidences R6G still to be present inside the AlO(OH) hollow spheres (Figure 21).

**Figure 22:**
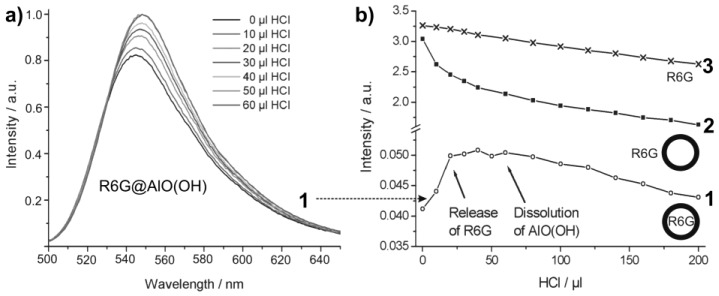
Course of the R6G photoluminescence intensity upon addition of hydrochloric acid: (1) R6G@AlO(OH); (2) AlO(OH) suspension with R6G added to the outside of the hollow spheres; (3) solution of R6G (modified reproduction from [55]). Only for (1) the complete sequence of emission spectra is shown; for (2) and (3) only the course of the integrated emission intensities is extracted and shown.

Controlled release of R6G from R6G@AlO(OH) was initiated by HCl-driven dissolution of the sphere wall. For this purpose, concentrated hydrochloric acid (32%) was added with a microliter injection syringe in portions of 10 µL to 4 mL of an aqueous suspension containing 8 mg of R6G@AlO(OH) hollow spheres. The course of the reaction was followed by photoluminescence upon 480 nm excitation (Figure 22a). Obviously, an increase in photoluminescence is observed up to an addition of about 30 µL HCl. This finding is in accordance to a certain absorption and reflection of the exciting as well as the emitted light due to the intact sphere wall. With proceeding dissolution of AlO(OH), R6G diffuses out of the now leaking hollow spheres and absorbs all incoming light. In sum, an increasing photoluminescence is observed then. Dynamic light scattering indeed proves a complete dissolution of the hollow spheres subsequent to an addition of about 60 µL of HCl [55]. During the acid-driven R6G-release, the pH-level of the suspension ranges from 8.2 [virgin AlO(OH) suspension] to 5.8 (release of R6G completed) and is then further decreased with proceeding formation of the acidic complex [Al(H_2_O)_6_]^3+^.

For further validation of the R6G-release, the integrated photoluminescence intensity of R6G@AlO(OH) (1) is compared to two references (Figure 22b): (3) an aqueous solution of R6G (10^−7^ M); (2) nonfilled AlO(OH) hollow spheres (2 mg/mL), which were suspended in aqueous R6G solution (10^−7^ M). The photoluminescence intensity in each case was calculated by mathematical fitting of the peak area of the relevant emission spectra (shown for (1) only). In contrast to R6G@AlO(OH) (Figure 22a), the emission for (2) and (3) decreases linearly with the addition of HCl. This is due to well-kown acidic quenching of R6G photoluminescence [78]. With R6G released out of the AlO(OH) sphere, quenching was also found for (1). When adding more than 60 µL of HCl, the photoluminescence of (1) is finally in parallel to (2) and (3). Both effects—the increase in photoluminescence for (1) and the significantly different behaviour in comparison to (2) and (3) —clearly evidence the container-type functionality of R6G@AlO(OH).

**Figure 23:**
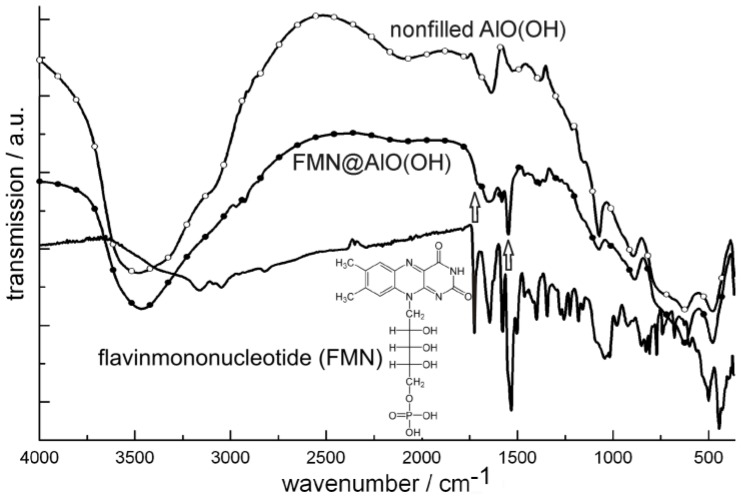

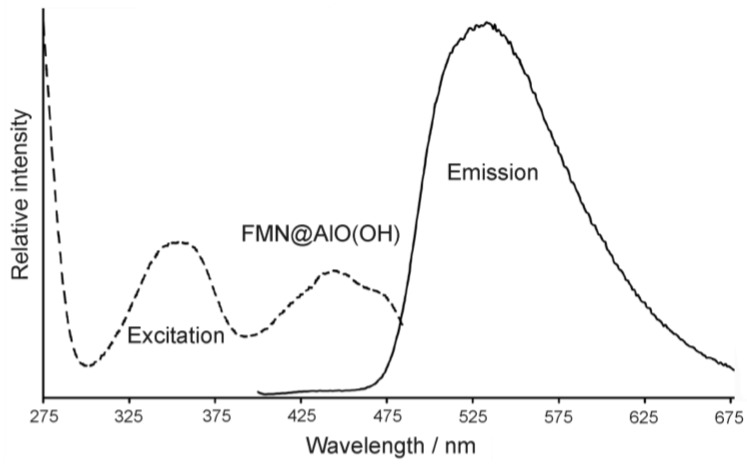
FT-IR spectra of FMN@AlO(OH) (vibrations of FMN indicated by arrows; nonfilled AlO(OH) hollow spheres and NaH(FMN) as references) as well as excitation and emission spectra of FMN@AlO(OH) (λ_emission_ = 500 nm, λ_excitation_ = 350 nm).

As a second fluorescent dye, flavinmononucleotide (FMN)—a derivative of vitamin B2—was encapsulated in AlO(OH) [77]. Here, 50 mg of FMN were encapsulated in about 120 mg of AlO(OH) hollow spheres. The successful encapsulation was again evidenced by spectroscopy. Thus, FT-IR spectra of flavinmononucleotide-filled hollow spheres [FMN@AlO(OH)] are compared in Figure 23 to nonfilled AlO(OH) hollow spheres and NaH(FMN) as references. 

Accordingly, the broad absorption of AlO(OH) is superpositioned by the strongest vibrations of the fluorescent dye, which indicates the presence of FMN. Moreover, FMN@AlO(OH) exhibits intense emission of green light upon excitation with UV-light or a blue-emitting LED (Figure 23). The resulting excitation and emission spectra are similar to those of the non-encapsulated fluorescent dye [79].

## 5. Perspectives of Nanoscale Hollow Spheres

The microemulsion approach opens up a broad access to nanoscale hollow spheres that are composed of various materials. These include metals (e.g., Ag, Au), as well as metal oxides [e.g., ZnO, TiO_2_, SnO_2_, AlO(OH), La(OH)_3_] and metal sulfides (e.g., Cu_2_S, CuS). Controlled adjustment of sphere diameters (*i.e.*, 10−60 nm) and fine-tuning of the inner cavity (*i.e.*, 2−30 nm) is possible, too. Profound characterization of hollow spheres requires the use of different and independent analytical tools with electron microscopy in all its variations as the most important one. Besides the variety of materials that come into range, the microemulsion approach is especially beneficial when aiming at container functionalities of hollow spheres. Encasulation of selected inorganic salts (e.g., KSCN, K_2_S_2_O_8_, KF), biomolecules and bioactive molecules (e.g., phenylalanine, quercetine, nicotinic acid) and fluorescent dyes (e.g., rhodamine, riboflavin) impressively prove this aspect.

**Figure 24:**
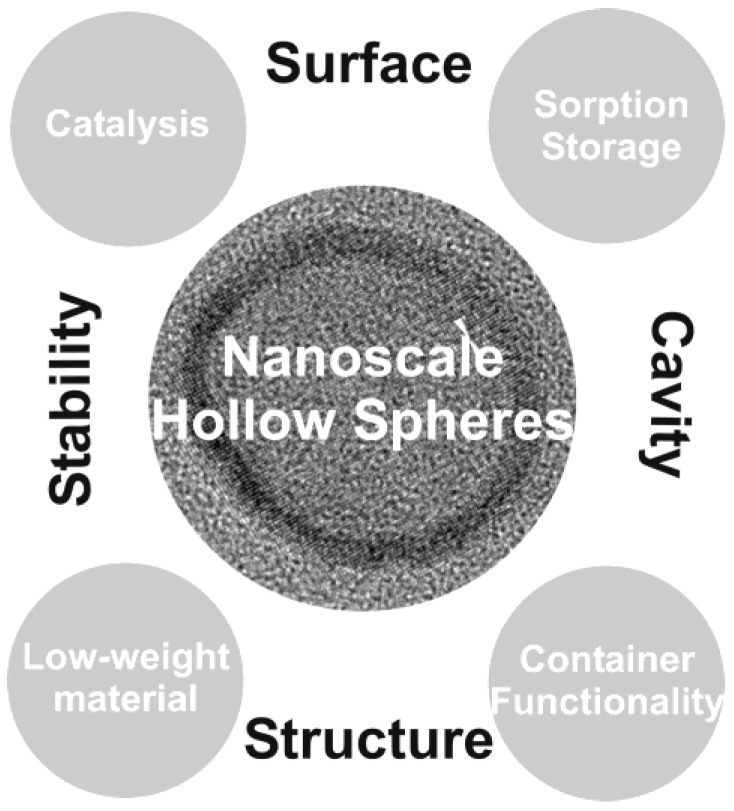
Future perspectives of nanoscale hollow spheres: properties and potential areas of application.

Nanoscale hollow spheres—no matter what the actual strategy of preparation might be—as a class of material with a special morphological structure will be of growing interest with regard to properties and application. Some of the most fascinating aspects are displayed in Figure 24. Thus, the large surface of nanoscale hollow spheres may lead to novel materials for catalysis, sensors, high-energy-density batteries as well as materials for gas sorption, gas separation and storage. In comparison to massive nanoparticles the mechanical and thermal stability of hollow structures can be beneficial due to reduced thermal sintering—and thereby a less reduced specific surfaces—at the elevated temperatures (200−500 °C) that are often required for device operation. Additional flexibility may arise from the fact that nanoscale hollow spheres can be modified and coated differently on their inner and outer surface. The combination of high mechanical stability and low specific weight may lead to low-weight building materials. Finally, the inner cavity opens up options to all kinds of container functionalities. To this concern, application in molecular biology and medicine might be most challenging. This includes controlled transport and release of, for instance, tumor agents, analgetics, inflammation retardants or hormones. Hollow spheres may also act as contrast agents for modern imaging techniques such as ultrasound imaging (US) or magnetic resonance tomography (MRT). Despite from biomedical issues, however, container functionalities are also promising for controlled release of anti-oxidants, lubricants or electrolytes.

Altogether, many features of nanoscale hollow spheres such as diffusion and mass transport through the sphere wall, mechanical stability, magnetic and conductive properties are yet right at the beginning of fundamental exploration and research. Albeit—A wide variety of materials with precise control of size and composition is required to go for the above properties and applications. The microemulsion approach as presented here will surely be a route to do so.
